# The Legislation of 1902

**Published:** 1901-12-28

**Authors:** 


### The Hospital

A Journal of

The Medical Sciences and Hospital Administration.

Vol. XXXI.—No. 796.] Edited by Sir Henry Burdett, K.C.B., and Solomon C. Smith, M.D., M.R.C.P. [December 28, 1901.

### The Legislation of 1902.

If we look at the coming session even from a purely medical point of view, we cannot fail to perceive a considerable number of openings for profitable effort; some of them in relation to the internal affairs of the profession, others in relation to its position and duties in the State. On nearly all such matters, however, there are possibilities of taking different or even opposite points of view; even if only those which would be selected by complete knowledge on the one hand and by absolute ignorance on the other. In such conditions as these, it was once thought to be the province of governments to govern; or, in other words, to take into account the representations of those who entertained discordant views upon any subject of public importance, and to decide to what extent these views should respectively prevail. In our day, however, and by virtue of the gradual disappearance of statesmen to make way for politicians, we have changed all that; and it is regarded as the first duty of a minister, or at least as the first after he has made adequate provision for the members of his own family, to avail himself of the absence of unanimity among reformers as a valid excuse for leaving all evils unreformed. The expectations held out by politicians, when contrasted with the amount of realisation by which they are ordinarily succeeded, have often reminded us of the stock in trade of certain itinerant merchants, often to be seen in second-rate thoroughfares, who walk about under huge clusters of bags of indiarubber, gaily coloured, and inflated with air to their utmost limits of expansion. The merchants exchange these bags for bones, old bottles, rags, and such like material, brought to them by the ingenuous youth of the localities which they frequent; nay, in some cases even for actual coined money of the realm; and the purchasers, whenever their prizes are exposed to so much as a pin prick, find themselves possessed only of a scrap of shrivelled membrane, a pinch of coloured powder, and a bad smell. In the political market, the analogues of these bags are commonly known as "judicious compromises."

In the eyes of any government which had attained to a proper sense of its duties and responsibilities towards the country, there could be few questions more imperatively calling for a settlement than that of the education and registration of midwives. Coroners, all over England, have for years borne ghastly testimony to the fact that maternal life is largely and continuously sacrificed by the ignorance of many of the women who undertake to officiate in this capacity; and, on the principle that the first duty of a government is to protect the lives and property of the governed, there can be no doubt that something should long ago have been done in the direction of at least an endeavour to remedy the evils complained of. Certain philanthropists have brought forward their scheme, and have more than once succeeded in introducing it within the doors of Parliament. Objectors, chiefly within the ranks of the medical profession, have not been wanting; and the scheme has been exposed to criticism which has fully shown that its details require careful consideration. Such consideration it was the plain duty of the Government to give, and by means of it to construct a measure which should aim at combining the best possible protection to lying-in women, with the smallest attainable injury to medical practitioners. The only thing done in this direction has been an appeal by the Privy Council to the Medical Council, followed by an immediate and unblushing abandonment of the duty which not only existed, but which the very appeal itself had recognised as a reality. The excuse, of course, was the "difficulty" of carrying a proper measure through Parliament, and it is hard to imagine a more pitiable one. The business of a government is to overcome the difficulties incidental to the task of governing; and one that will not take the trouble to do this could surely find no greater difficulty than that of justifying its own existence. Besides legislation with regard to midwifery, it is urgently required in respect of other matters which have important bearings upon public health; such, for example, as the fraudulent practice of medicine or dentistry by joint stock companies, the existing state of the law with regard to the certification and registration of deaths, the conditions under which charges upon local rates for drainage and other sanitary purposes are at present authorised, perhaps even an amendment of what may be collectively described as medical legislation, including, of course, the constitution and powers of the Medical Council. All these issues involve matters of much concern, and are calculated to tell seriously not only upon the general death-rate of the country, but also upon the efficiency of many of those who abstain from swelling it. Governments are paid, in money, power, and patronage, in order that they may take action in such matters, and not at all in order that they may find excuses for inaction. To say that the domestic legislation of the country must remain unattended to, because we are engaged in war in another continent, is simple nonsense.

